# Insulin Treatment Does Not Prevent EARLY Autonomic Cardiovascular and Diastolic Dysfunctions in Streptozotocin-Induced Diabetic Rats †

**DOI:** 10.3390/ph17050577

**Published:** 2024-04-30

**Authors:** Sarah C. F. Freitas, Marina R. H. Dutra, Paulo M. M. Dourado, Victor Hugo de Martins Miranda, Camila P. dos Santos, Iris C. Sanches, Maria-Cláudia Irigoyen, Kátia De Angelis

**Affiliations:** 1 Translational Physiology Laboratory, Universidade Nove de Julho (UNINOVE), São Paulo 01525-000, Brazil; marinarhdutra@gmail.com; 2 Hypertension Unit, Heart Institute (InCor), School of Medicine, University of São Paulo (USP), Sao Paulo 05403-000, Brazil; pmdourado@terra.com.br (P.M.M.D.);; 3 Department of Physiology, Federal University of Sao Paulo (UNIFESP), São Paulo 04023-062, Brazil; vhmmiranda85@gmail.com (V.H.d.M.M.);; 4 Movement Laboratory, Sao Judas Tadeu University (USJT), Sao Paulo 03166-000, Brazil

**Keywords:** diabetes, glycemic control, cardiac dysfunction, autonomic cardiovascular dysfunction, insulin replacement therapy, inflammation

## Abstract

Recent studies have found increased cardiovascular mortality risk in patients with type 1 diabetes when compared to normoglycemic people, even when they were kept under good glycemic control. However, the mechanisms underlying this condition have yet to be fully understood. Using streptozotocin (STZ)-induced diabetic rats, we evaluated the effects of insulin replacement therapy on cardiac, autonomic, inflammatory, and oxidative stress parameters. Daily treatment with insulin administrated subcutaneously in the STZ-diabetic rats showed a reduction in hyperglycemia (>250 mg/dL) to normalized values. The insulin treatment was effective in preventing alterations in cardiac morphometry and systolic function but had no impact on diastolic function. Also, the treatment was not able to prevent the impairment of baroreflex-tachycardic response and systolic arterial pressure variability (SAP-V). A correlation was found between improvement of these autonomic parameters and higher levels of IL-10 and lower levels of oxidized glutathione. Our findings show that insulin treatment was not able to prevent diastolic, baroreflex, and SAP-V dysfunction, suggesting an outstanding cardiovascular risk, even after obtaining a good glycemic control in STZ-induced diabetic rats. This study shed light on a relatively large population of diabetic patients in need of other therapies to be used in combination with insulin treatment and thus more effectively manage cardiovascular risk.

## 1. Introduction

Cardiovascular diseases are the main cause of death worldwide, and diabetic patients are more prone to such events than the rest of the population. In fact, it is well established in the literature that diabetes mellitus (DM) is associated with a higher risk of cardiovascular diseases. Malmberg and collaborators have demonstrated that diabetic patients with no previous cardiovascular disease have the same long-term morbidity and mortality of nondiabetic patients with a previous cardiovascular event [1]. Additionally, there is a higher risk of cardiovascular mortality associated with type 1 diabetes than in the general population [2]. Insulin therapy is the most used treatment for type 1 DM patients, and it has the goal of reaching normal values of glycated hemoglobin (HbA1c, <7%). However, recent studies have shown that even after achieving this excellent threshold of HbA1c, these diabetic patients are still at increased cardiovascular risk when compared to their normoglycemic peers. Studies have found that they have a more than two-fold increased risk of overall mortality and an approximately threefold increased risk of cardiovascular disease-associated mortality [3,4].

It is now well-established that changes in glucose metabolism represent an independent risk factor for cardiovascular disease, even with increased blood glucose levels below the diabetes threshold [5]. In this sense, hyperglycemia may be viewed as a major factor for the onset of diabetes complications. High glucose may induce many detrimental factors, mainly at the microvasculature level, leading to the development of chronic complications of diabetes (such as cardiomyopathy, nephropathy, and neuropathy) by causing increased oxidative stress, inflammation, and apoptosis, which may result in tissue remodeling in several organs [6]. In diabetic patients, autonomic neuropathy is first characterized by an impairment in the vagal nerve, which leads to an imbalance of parasympathetic and sympathetic activities in the heart [7]. The streptozotocin (STZ)-induced type 1 DM in rats is a widely used experimental model of diabetes and has been found to be particularly useful in the study of the disease and the effects of chronic hyperglycemia. In fact, in a type 1 DM experimental model, it has been already demonstrated that, after 3 days of STZ induction of hyperglycemia in rats, they presented autonomic dysfunction [8]. STZ-induced diabetic rats exhibit cardiac alterations similar to the ones clinically observed in diabetic patients, such as alterations in cardiac morphometry, systolic and diastolic function [9], reduction of cardiac contractility [10], impaired autonomic function, and baroreflex sensitivity [11].

Although the role of hyperglycemia in diabetic-induced complications has yet to be fully understood, cardiac autonomic dysfunction has been found to be an important predictor of higher mortality in diabetic patients [12], while the autonomic nervous system plays a key role in regulating inflammation and oxidative stress in different tissues and conditions [13]. Hence, the STZ diabetic model is quite adequate for determining the role of insulin treatment, preventing the alterations related to the hyperglycemic cardiovascular complications. This model also helps to shed light on what mechanisms can still be changed and accounts for the high cardiovascular risk observed in diabetic patients, even after achieving excellent glycemic control [3,4]. Therefore, we tested insulin as a cardiovascular neuromodulator therapy in an animal model of type 1 diabetes. We hypothesized that, even after the normalization of blood glucose levels, cardiac abnormalities are still present in this model of STZ-induced type 1 DM. The study of the mechanisms involved in diabetic cardiovascular dysfunctions, as inflammatory and oxidative stress markers, may help to explain the higher mortality in the diabetic population even under good glycemic control with insulin treatment.

## 2. Results

### 2.1. Glycemia, Insulin Levels, and Body Mass Evaluations

Two days after the diabetes induction, glycemia was measured in all rats. Hyperglycemia was found in the diabetic groups (>250 mg/dL). However, while the D group remained with elevated glycemia during the 30 days, the treatment with insulin in the DI group was effective in normalizing the glycemic values of these animals over the four weeks of the protocol (Table 1).

At the end of fourth week of protocol, the DI group presented higher insulin levels than the D group and similar levels of insulin when compared to the C group (Table 1).

Since the first week, the D group showed a reduction in body mass when compared to the C and DI groups. The insulin treatment was able to maintain body mass in the DI group, presenting similar values to those observed in the C group over the 4-week protocol (Table 1).

### 2.2. Echocardiography

#### 2.2.1. Cardiac Morphometry

**Figure 1:**
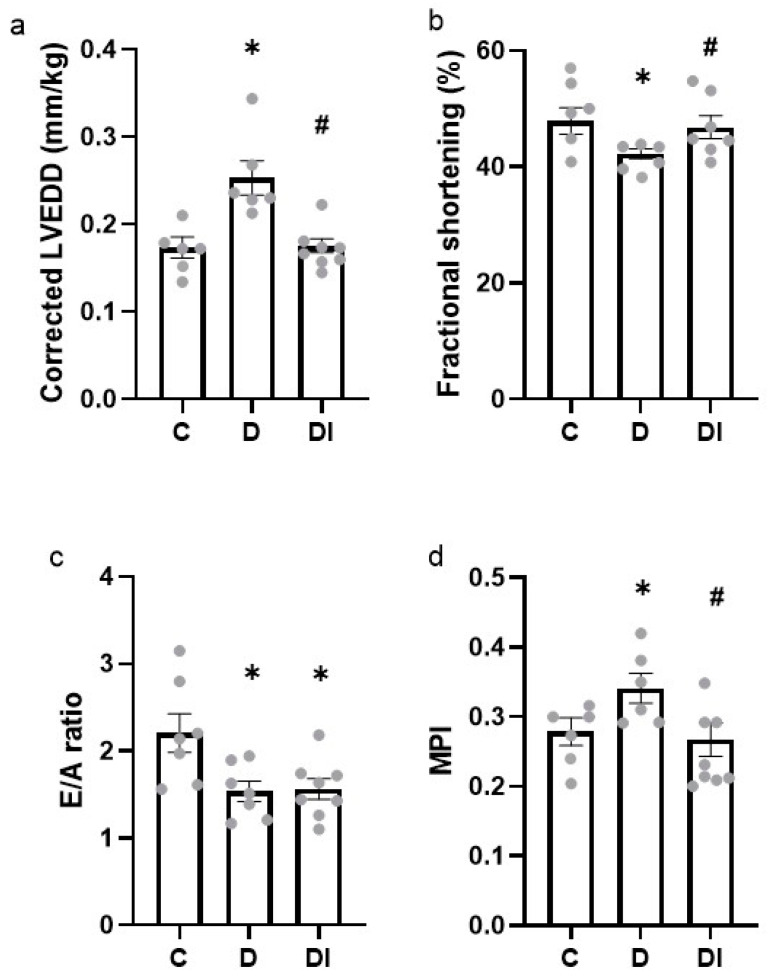
Echocardiographic evaluations. (**a**) Left ventricle end-diastolic diameter (LVEDD) corrected by body mass, (**b**) E-wave and A-wave ratio, (**c**) fractional shortening, and (**d**) myocardial performance index (MPI). Mean ± SEM, analyzed using one-way ANOVA followed by post hoc Student–Newman–Keuls test; *n* = 6–8/group; * *p* < 0.05 vs. C and ^#^ *p* < 0.05 vs. D; C: control group, D: diabetic group and DI: diabetic group treated with insulin.

The LV end-diastolic diameter (LVEDD) was not different between groups in absolute value, but when corrected by body mass, an increase in the D group was observed, but this was prevented in the DI group (Table 2 and Figure 1a, respectively).

Diabetic animals also presented a reduction in left ventricular posterior wall thickness at diastole (LVPWd) and in relative wall thickness (RWT); these changes were prevented by the insulin treatment. Thus, the LV mass in the D group was decreased, and treatment with insulin was also able to prevent this alteration (Table 2).

#### 2.2.2. Systolic Function

There was no difference in the ejection fraction between the groups (Table 2). There was a reduction in fractional shortening in the D group, which was prevented by insulin treatment in the DI group (C: 48 ± 1.7; D: 41 ± 1.3 and DI: 47 ± 1.9%) (Figure 1b). The cardiac output and the stroke volume were higher in the DI group compared to the D groups (Table 2).

#### 2.2.3. Diastolic Function

The isovolumic relaxation time (IVRT) in absolute value was not different between the groups; however, the IVRT normalized by the RR interval and was increased in the D group and in the DI group when compared to control animals (Table 2). Similarly, the E/A ratio was decreased in the D group, and the insulin treatment was also not able to prevent this dysfunction (Figure 1c).

The peak E-wave and A-wave velocity did not present any difference when the three groups were compared (Table 2). However, the deceleration time of the E wave in absolute value was increased in both the D and DI groups, while no difference was found for the normalized values by the RR interval (Table 2).

#### 2.2.4. Global Function

The global function was assessed using the myocardial performance index (MPI); this parameter was increased in the D group, and this alteration was prevented in the DI group (Figure 1d).

### 2.3. Hemodynamic and Baroreflex Sensitivity Assessment

The MAP, as well as the SAP and diastolic AP (DAP), were reduced in D group when compared to C and DI group, showing that the insulin treatment was effective in preventing AP alterations (Table 3). The same response was observed for HR, and the reduction in the D group was avoided in the DI group, presenting similar values to those of the C group (Table 3).

**Figure 2:**
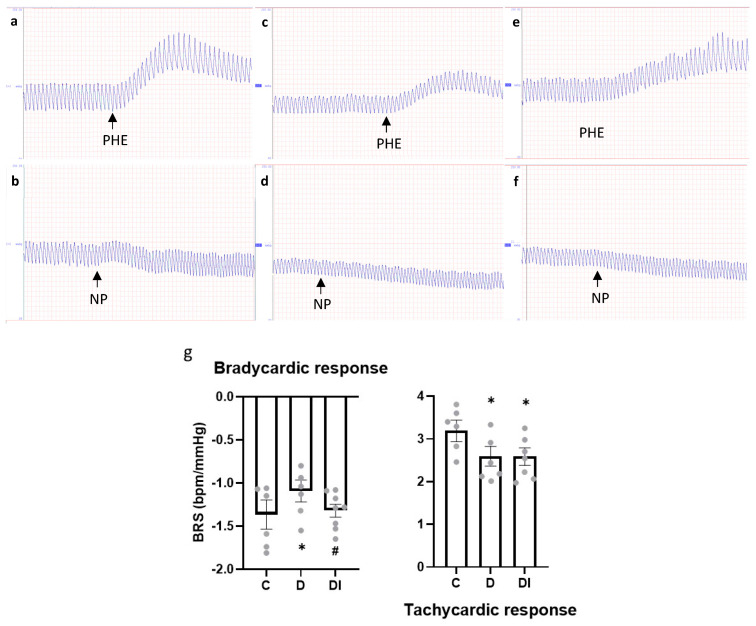
Baroreflex sensitivity (BRS) evaluations. Original recording of bradycardic response to phenylephrine injection (PHE) and tachycardic response to sodium nitroprusside injection (NP) in (**a**,**b**) control animal, (**c**,**d**) diabetic animal, and (**e**,**f**) diabetic treated with insulin animal, respectively. (**g**) BRS index for bradycardic and tachycardic responses. Mean ± SEM; analyzed using one-way ANOVA, followed by post hoc Student–Newman–Keuls test; *n* = 6–8/group; * *p* < 0.05 vs. C and ^#^ *p* < 0.05 vs. D; C: control group, D: diabetic group and DI: diabetic group treated with insulin.

Baroreflex sensitivity, as assessed with bradycardic response, showed a decrease in the D group, and insulin treatment was effective in preventing this dysfunction. However, the tachycardic response was reduced in the D group, and the decrease remained even with the insulin treatment in the DI group (Figure 2).

### 2.4. Autonomic Control of Heart Rate

**Figure 3:**
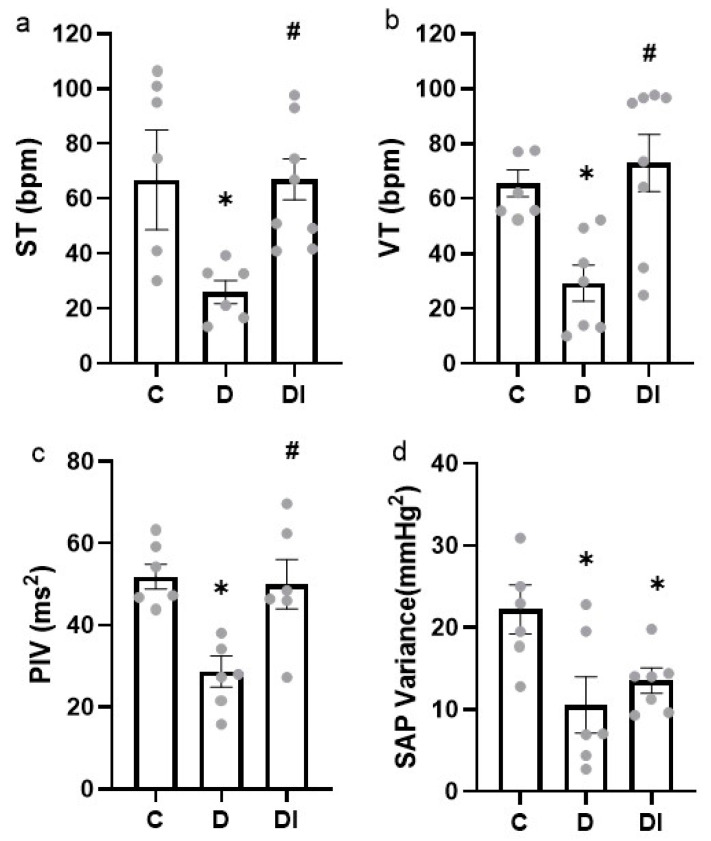
Autonomic control and modulation of the heart (**a**) Sympathetic tone (ST), (**b**) Vagal tone (VT), (**c**) Pulse interval variability (PIV) and (**d**) Systolic arterial pressure (SAP) variability. Mean ± SEM; analyzed by one-way ANOVA, followed by post hoc Student-Newman Keuls test; *n* = 6–8/group; * *p* < 0.05 vs. C and ^#^ *p* < 0.05 vs. D; C: control group, D: diabetic group and DI: diabetic group treated with insulin.

The diabetic animals showed reduced cardiac sympathetic and vagal tone (ST and VT), but this decrease was prevented in diabetic animals treated with insulin (Figure 3a,b).

Intrinsic HR, assessed using a cardiac autonomic double blockade, was decreased in the D group, and insulin treatment was able to prevent it in the DI group (D: 339 ± 6 vs. C: 388 ± 7 and DI: 405 ± 9 bpm).

### 2.5. Cardiovascular Autonomic Modulation

The diabetic group showed a reduced PIV (Figure 3c), and the insulin treatment was able to maintain this parameter at the control values. The RMSSD, another time-domain parameter evaluated, was not different between the studied groups (C: 5.82 ± 0.39; D: 7.02 ± 0.19 and DI:6.67 ± 0.38 ms). In frequency domain analysis, the LF and HF of the PI showed no significant differences between the groups (LF—C: 2.46 ± 0.82; D: 1.13 ± 0.19 and DI: 3.21 ± 0.67 ms^2^; HF—C: 7.53 ± 1.48; D: 10.53 ± 0.85 and DI: 10.07 ± 0.54 ms^2^).

In the evaluation of the SAP variability parameters, the decrease in the D group was not prevented by insulin treatment and was still altered in the DI group when compared to the C group (Figure 3d). The LF of the SAP showed no difference between the studied groups (C: 1.79 ± 0.22; D: 2.44 ± 0.93 and DI: 2.67 ± 0.41 mmHg^2^).

### 2.6. Inflammatory Mediators

**Figure 4:**
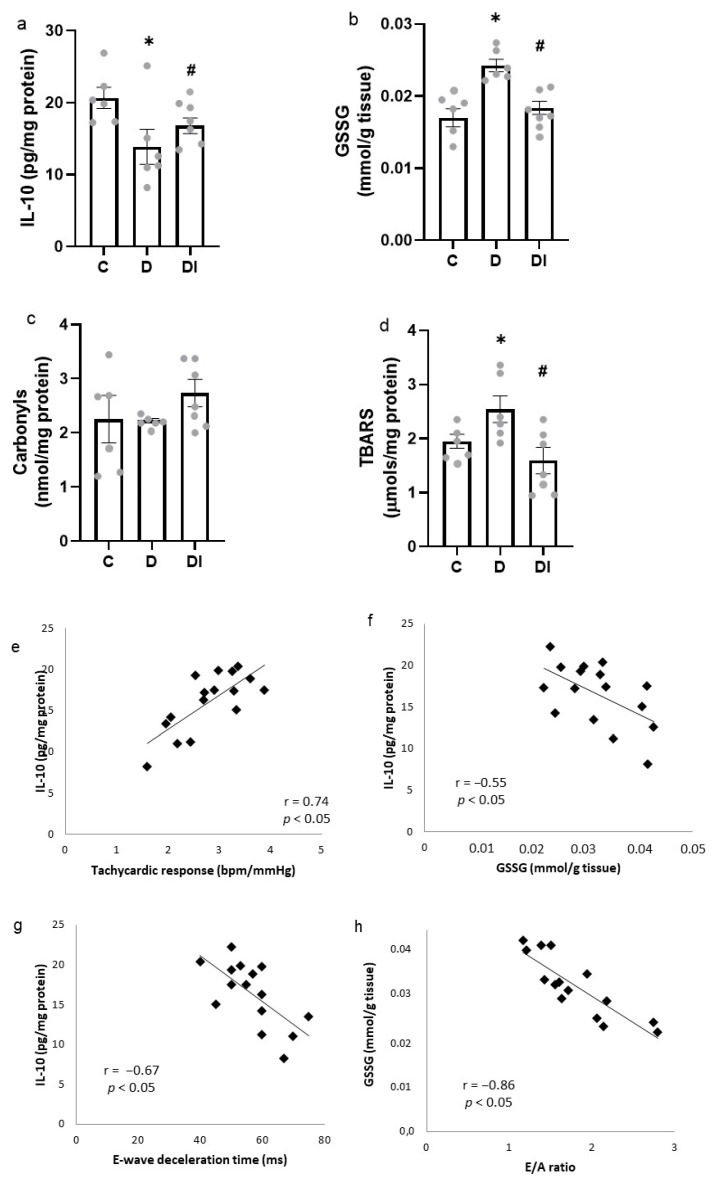
Anti-inflammatory and oxidative stress biomarkers in left ventricle. Evaluations of (**a**) IL-10, (**b**) oxidized glutathione (GSSG), (**c**) oxidized proteins (carbonyls), (**d**) thiobarbituric acid reactive substances (TBARS). Mean ± SEM, analyzed using one-way ANOVA followed by post hoc Student–Newman–Keuls test; *n* = 6–8/group; * *p* < 0.05 vs. C and ^#^ *p* < 0.05 vs. D; C: control group, D: diabetic group and DI: diabetic group treated with insulin. Pearson correlation between (**e**) interleukin-10 (IL-10) and tachycardic response, (**f**) IL-10 and oxidized glutathione (GSSG), (**g**) IL-10 and E-wave deceleration time, (**h**) GSSG and E/A ratio (*n* = 5–6/group).

The IL-10 evaluation showed a reduction of this cytokine in the diabetic group, which was prevented by insulin treatment in the DI group (Figure 4a). TNF-α and IL-6 measurements did not present any difference between the studied groups (TNF-α—C: 8.13 ± 0.68; D: 7.03 ± 0.49; DI: 7.16 ± 0.84 and IL-6—C: 138.06 ± 11.29; D: 120.21 ± 10.80; DI: 154.22 ± 10.04 pg/mg protein).

### 2.7. Oxidative Stress Analysis

Lipoperoxidation measured by TBARS demonstrated an increase in the D group, and the insulin treatment was effective in preventing this alteration in the DI group (Figure 4b). The values obtained for the oxidized proteins by carbonyls were not different among the three groups (Figure 4c).

The glutathione (GSH) total in the left ventricle was increased in the D group when compared to the others (GSH total—D: 0.452 ± 0.040 vs. C: 0.293 ± 0.019 and DI: 0.320 ± 0.014 mmol/g tissue). The reduced/oxidized glutathione (GSH/GSSG) ratio was similar between groups (GSH/GSSG ratio—C: 16.55 ± 0.94; D: 16.46 ± 0.78 and DI: 16.51 ± 0.63). The diabetic group showed an increase in both reduced glutathione and oxidized glutathione. The DI group preserved these glutathione levels when compared to control values (Figure 4d).

The CAT and GPx activities, two antioxidant enzymes, were higher in the diabetic group, and their activities in the DI group were maintained at control levels (D: 1.32 ± 0.08 vs. C: 0.81 ± 0.05 and DI: 0.79 ± 0.05 nmol/mg protein; D: 0.081 ± 0.007 vs. C: 0.048 ± 0.011 and DI: 0.048 ± 0.004 nmol/min/mg protein, respectively).

There was an increase in TRAP, a non-enzymatic antioxidant measure, in both the D and DI groups when compared to the C group (D: 5.64 ± 0.91 and DI: 4.84 ± 0.58 vs. C: 2.73 ± 0.41 µM of Trolox).

### 2.8. Pearson Correlation

Positive correlations were found between the tachycardic response of baroreflex and IL-10 (r = 0.74, *p* < 0.05; Figure 4e) and between the SAP variance and IL-10 (r = 0.59, *p* < 0.05). The increase in SAP variance (normalization) and IL-10 levels were both correlated with the reduction in oxidized glutathione (r = −0.73, *p* < 0.05 and r = −0.55, *p* < 0.05; Figure 4f, respectively).

Diastolic function parameters were also associated with inflammation and oxidative stress. The increase in E-wave deceleration time was correlated with a reduction in IL-10 (r = −0.67, *p* < 0.05; Figure 4g), and the E/A ratio increase was correlated with diminished oxidized glutathione (r = −0.85, *p* < 0.05; Figure 4h).

## 3. Discussion

Several studies have documented higher mortality in the diabetic population, particularly from cardiovascular events, along with a shorter life span and higher mortality risk from ischemic heart disease in the type 1 diabetic population [2,14]. Furthermore, recent research has demonstrated that, regardless of glycemic control, the mortality rate remains higher in the type 1 DM population when compared to their age-matched controls without diabetes [4,15]. A study has recently reported that type 1 DM patients with HbA1c levels of 6.9% or lower had a threefold risk of cardiovascular mortality when compared to controls [3]. Given this scenario, we tested the potential of insulin therapy as a cardiovascular neuromodulator in a model of type 1 diabetes. The most important finding of this article lies in the fact that, despite the beneficial effects of insulin treatment on glycemic and insulin levels, body mass and other clinical variables such as treatment was not able to prevent the diastolic dysfunction and impairments in baroreflex sensitivity and in the SAP variance in the STZ-induced diabetic animals.

An STZ-induced diabetes model is largely used to study cardiovascular and autonomic type 1 diabetes consequences. In this sense, findings from previous studies have demonstrated a range of responses to insulin treatment in reversing/attenuating cardiac dysfunctions in STZ experimental diabetes. This may be due to the species used, STZ dosage, type of insulin treatment, the time course of insulin treatment, and glycemic variability during the day [16,17,18,19]. However, few studies have focused on the role of insulin treatment in preventing these alterations, instead of reversing them. Thus, considering a translational point of view that insulin treatment is still the main form of treatment for type 1 diabetic patients, we investigated the treatment with insulin and normalization of glycemic levels, as recommended to type 1 diabetic patients, starting immediately after diagnose [20] and aiming to improve glycemic control and preventing diabetic-associated dysfunctions. In this sense, our findings demonstrated that the DI group had a normalization of glycemia and a prevention of body mass loss since the first week of the protocol.

Moreover, diabetic cardiomyopathy, clinically characterized by early diastolic dysfunction, followed by ventricular remodeling and, at later stages, systolic dysfunction [21] was attenuated in insulin-treated STZ-diabetic rats. In fact, treatment approaches are indeed relevant in the diabetic population, since the majority of patients with well-controlled diabetes and the absence of other cardiovascular comorbidities exhibit impaired diastolic function (up to 75% of people with diabetes) [22]. In this sense, our findings showed that the D group developed characteristics of a dilated cardiomyopathy, with increased LVEDD and decreased LVPWd and RWT, accompanied by diastolic and systolic function impairment. The DI group maintained cardiac morphometry and systolic function at control levels, although it presented increased IVRT, decreased E/A ratio, and increased E-wave deceleration time, showing that treatment with insulin was not able to prevent changes in diastolic function, even after the improvement of glycemic control. Other studies with insulin treatment in STZ-induced diabetic animals, starting from five days, one week, and even five months of diabetes induction, have shown conflicting results, with some demonstrating partial and others full recovery of cardiac systolic and diastolic dysfunctions [17,18,19] or neither of them [16]. However, the treatment in those cases started only when cardiovascular and autonomic complications in these animals were well-established [9,23].

The improved glycemic control achieved was also able to prevent both AP and HR alterations in this model [10,11]. Moreover, another beneficial effect of insulin may be seen by the prevention of the cardiac autonomic control/modulation dysfunction observed in type I diabetic patients. In STZ-diabetic rats, there is, initially, a reduction of the parasympathetic tone, followed by impairment in cardiac sympathetic tone and in PIV [23,24,25]. Our findings demonstrated that treatment with insulin may preserve cardiac vagal and sympathetic tonus, as well as PIV. However, insulin treatment was not able to prevent changes in AP variability and baroreflex sensitivity, two important mechanisms responsible for AP regulation, and was found to be autonomic function markers [26]. The tachycardic response of baroreflex and SAP variance remained lowered in STZ-induced diabetic even after insulin treatment. In fact, it was reported that STZ-induced diabetic rats present a reduction of tachycardic and bradycardic baroreflex sensitivity and AP variance [11,25,27], as we found in the D group of the present study. The reduction observed in SAP variance of both the D and DI groups in our research may indicate a reduced vascular autonomic modulation, which is prone to decrease when there are high levels of sympathetic autonomic activity. It should be emphasized that SAP variability impairment is associated with target organ damage, as recently reviewed by our group [28].

It is known that diabetes complications in patients and animal models are related to inflammation and oxidative stress. In fact, we observed a reduction in IL-10 levels in the diabetic group when compared to both C and DI groups, although no difference in TNF-α and IL-6 levels was observed between the groups. The IL-10 levels in serum was associated with parasympathetic activity in the heart [29]. Moreover, our findings showed that higher IL-10 levels were correlated with improved tachycardic response sensitivity and SAP variance and lower IL-10 levels with a higher E-wave deceleration time. Similarly, a correlation was also observed in animals with higher GSSG, as they had a lower E/A ratio. Thus, these correlations suggest the animals with lower IL-10 or higher oxidized glutathione had a more severe diastolic dysfunction.

In this sense, when we analyzed oxidative stress in the LV, the diabetic group presented a significantly augmented activity of antioxidant enzymes, seen by CAT and GPx. This may explain the absence of alteration in protein oxidation by carbonyls between the groups, although the D group presented elevated lipoperoxidation by TBARS, even with increased adaptation of the antioxidant response. Insulin treatment restored all these oxidative stress biomarkers. TRAP, which measures non-enzymatic antioxidants, was increased in D group and was still augmented in DI group, when compared to C group. In fact, the antioxidant adaptation towards protective cardiac tissue in STZ-induced diabetic rats was already described in another study [30], as well as an impaired autophagy process in STZ-induced diabetic animals has been observed [31], which may be related to the antioxidant adaptation, since autophagy dysfunction leads to augmented antioxidant response [32].

The glycemic control achieved here was enough to normalize the weekly glycemia and body mass, and the insulin levels at the end of the protocol. The measurement of HbA1c levels is a limitation of the study, however research has pointed that levels of HbA1c are not good markers of glycemic variability along the day in patients, accounting for only a 25% risk of further complications [33]. In this sense, it is believed that the maintenance of the alterations reported may be due to hypoglycemic and hyperglycemic peaks occurring throughout the day. In fact, hyperglycemic peaks seems to lead to increased epigenetic changes and induce greater inflammation than in continuous levels [33]. It is important to point that isoflurane is a highly suitable choice of anesthetic for echocardiogram evaluation. However, the selection of ketamine and xylazine as the anesthetics for this evaluation underwent careful consideration in accordance with our study sequential procedures. This consideration was particularly crucial as the animals transitioned from echocardiogram analysis to cannulation surgery under the same anesthetic regimen. After a 24-h interval post-administration of the anesthetic, blood pressure recordings were conducted under awake conditions. Importantly, all experimental groups adhered to identical anesthesia protocols, HR was carefully monitored, and subsequent analyses of echocardiographic parameters, blood pressure, and heart rate confirmed values previously reported in controls and STZ rats [9,10,11,12,18]. In addition, further studies should be aimed at assessing other metabolic variables, such as free fatty acids and triglycerides, and investigate whether metabolic changes caused by hyperglycemia are preventable with insulin treatment, after a normalized glycemic level is achieved. Moreover, exploring adjunctive therapies such as antioxidants or exercise is crucial to corroborate the potential underlying mechanisms contributing to the early dysfunctions observed in this diabetic model. Another limitation of the study is that the residue defects of STZ-induced diabetic model after the insulin treatment may potentially result from either diabetes or the previously unattended direct effects of STZ on non-beta cells; hence, further studies using a different model of diabetes are warranted. Finally,

## 4. Materials and Methods

### 4.1. Animals

Male Wistar rats (230–260 g) were obtained from Universidade Nove de Julho. They received standard chow and water ad libitum. They were housed into boxes containing 3–4 animals/each to 22–24 °C with day/night cycle of 12 h. The investigation conforms with the principles outlined in the Declaration of Helsinki, the procedures and protocols followed the Guidelines for Ethical Conduct in the Care and Use of Animals approved by the Institutional Animal Care and Use Committee. This project was approved by the CEUA UNINOVE under registration number An0011/2014 and all surgical procedures and protocols were carried out in accordance with ARRIVE guidelines [34]. The animals were randomly assigned to one of the three groups (6–8/group): control group (C), diabetic group (D), diabetic group treated with insulin (DI). All the groups were followed for 30 days.

### 4.2. Diabetes Induction and Evaluation of Glycemia, Insulin Levels, and Body Mass

After fasting for six hours, the animals were randomly assigned to either diabetic groups or a control group, and diabetes was induced with a single injection of STZ (50 mg/kg; Sigma Chemical Co., St. Louis, MO, USA) in the tail vein, dissolved in a citrate buffer (0.01 M, pH 4.5) [35]. The control animals were injected with only a citrate buffer. Blood glucose was measured by hemoglucotest using test strips (Advantage Roche^®^, Mannheim, Germany) to confirm the glycemia at the beginning of the protocol and then measured weekly, and the assessments were performed in the afternoon, using an Accu-Check Instant test (Boehringer Mannheim, Mannheim, Germany). Measurements were undertaken without fasting, due to the risk of hypoglycemia in the animals treated with insulin. Animals presenting blood glucose above 250 mg/dL two days after the administration of STZ were selected for the diabetic groups and randomly distributed in the D and DI group. However, no formal method of randomization was used. Body mass was also measured weekly. All animals were monitored during the 30-day protocol, twice daily, and animal in the untreated diabetic group presented with polyuria e polydipsia. At the end of the protocol, insulin levels of plasma were measured with a commercially available ELISA kit (EZRMI-13K, Merck Millipore, Darmstadt, Germany).

### 4.3. Insulin Replacement

The animals in the DI group received daily doses of insulin, neutral protamine Hagedorn (NPH), subcutaneously, 2U in the morning and 4U in the afternoon [36]. The treatment began 2 days after STZ injection, immediately after the measurement of blood glucose for diagnosis of hyperglycemia and throughout the protocol (30 days of treatment).

### 4.4. Echocardiography

After 30 days of the protocol, echocardiographic evaluation was performed by a double blinded observer, following the guidelines of the American Society of Echocardiography. Rats were anesthetized (50 mg/kg ketamine and 10 mg/kg xylazine, i.p.), and the echocardiographic assessments were performed using Agilent, SONOS 5500 (Philips Medical Systems, Cambridge, MA, USA), and measured as previously described elsewhere [9].

### 4.5. Hemodynamic and Baroreflex Sensitivity Assessment

Twenty-four hours after the final echocardiographic evaluation, 2 catheters filled with saline were implanted into the carotid artery and jugular vein of the anesthetized rats (50 mg/kg ketamine and 10 mg/kg xylazine, i.p.). On the following day, the arterial cannula was connected to a strain-gauge transducer (Blood Pressure XDCR; Kent Scientific, Torrington, CT, USA), and the arterial pressure (AP) signals and pulse intervals (PI) were recorded over a 30-min period in conscious animals, as previously described [11]. Sequential bolus injections (0.1 mL) of increasing doses of phenylephrine (0.25–32 mg/kg) and sodium nitroprusside (0.05–1.6 mg/kg) were given to induce increases or decreases in mean AP (MAP) responses (for each drug), ranging from 5 to 40 mmHg. Baroreflex sensitivity was expressed as bradycardic response and tachycardic response in beats per minute per millimeter of mercury, as described elsewhere [11].

### 4.6. Autonomic Control of Heart Rate

After the baroreflex sensitivity assessment, the AP and PI were continuously recorded at a basal state and after intravenous injection of methylatropine (4 mg/kg, Sigma, Kawasaki, Japan; 0.2 mL). Because the HR response to these drugs reaches its peak within 3 to 5 min, this time interval was allowed to elapse before the heart rate measurement. Atenolol (8 mg/kg, Sigma; 0.2 mL) was intravenously injected 10 min after methylatropine, and again the response was evaluated after a simultaneous blockade with atenolol and methylatropine. On the following day, the sequence of injections was inverted (first atenolol and then methylatropine). The intrinsic heart rate (IHR) was evaluated after a simultaneous blockade with atenolol and methylatropine. The sympathetic tonus was determined as the difference between the maximum heart rate after the methylatropine injection and IHR. The vagal tonus was obtained by the difference between the lowest heart rate after the atenolol injection and IHR [37].

### 4.7. Cardiovascular Autonomic Modulation

The overall variability of the HR was assessed in the time and frequency domains by spectral estimation in the 30 min recorded basal period. Assessment in the frequency domain was carried out with the decomposition of the systogram using fast Fourier transform (FFT). After this mathematical remodeling, the absolute powers of the low frequency band were obtained (LF: 0.20–0.75 Hz), and the spectral power for the very-low-frequency (0.00–0.20) and high-frequency (HF: 0.75–4.0 Hz) bands were calculated with power spectrum density integration within each frequency bandwidth, using a specialized software (CardioSeries v1.0; DanielPenteado, Ribeirao Preto, Brazil) [38]. For these assessments, we used stable records, at least 5 min and a sampling frequency of 2000 Hz. Two components were also obtained from the spectral analysis. The LF component was used as an index of sympathetic activity. The HF component was used as an index of the parasympathetic activity. We also evaluated RMSSD (root mean square of the squares of the differences between successive normal RR intervals) and pulse interval variability (PIV) [39]. The time-frequency analysis was used to determine systolic arterial pressure (SAP) variability. The parameters for the time domain analysis consisted of calculating average values of SAP, and its variability was quantified by the variability of SAP. The variability of the pulse interval was obtained using the analysis of the tachogram obtained from the recordings of the SAP, in which the frequency of the beats was determined by the interval between the two systolic peaks.

### 4.8. Tissue Preparation

After the hemodynamic and autonomic analysis, the animals were euthanized by decapitation, under anesthesia (ketamine, 50 mg/kg, i.v.), and the left ventricle (LV) was homogenized. Immediately after removal, the LVs were rinsed in a saline solution, and trimmed to remove fat tissue and visible connective tissue. It was frozen in liquid nitrogen and kept in a freezer −80 °C for a maximum period of two weeks. For oxidative stress evaluations, the tissue was cut into small pieces, placed in an ice-cold buffer, and homogenized in an Ultra80 Turrax blender (UltraStirrer, Pilatusstrasse, Muri, Switzerland) with 1 g tissue per 4 mL 120 mM KCl and 20 nM sodium phosphate buffer, pH 7.4. The homogenate was centrifuged at 600× *g* for 10 min at −26 °C.

### 4.9. Inflammatory Mediators

A piece of LV was cut and homogenized for the evaluation of inflammatory mediators. Interleukin (IL)-6, IL-10, and tumor necrosis factor-α (TNF-α) levels were determined using a commercially available ELISA kit (R&D Systems, Minneapolis, MN, USA), following the manufacturer’s instructions. ELISA was performed in 96-well polystyrene microplates with a specific monoclonal antibody coating. The threshold of sensitivity for the IL-6, IL-10, and TNF-α assays was 15.0 pg/mL. The absorbance was measured at 540 nm in a microplate reader [40].

### 4.10. Oxidative Stress Analysis

#### 4.10.1. Thiobarbituric Acid Reactive Substances (TBARS) Assay

For TBARS, trichloroacetic acid (10%, *w*/*v*) was added to the homogenate to precipitate proteins and to acidify the samples. This mixture was then centrifuged (1000× *g*, 3 min), the protein-free sample was extracted, and thiobarbituric acid (0.67%, *w*/*v*) was added to the reaction medium. The tubes were placed in a water bath (100 °C) for 15 min. The absorbance was measured at 535 nm [41].

#### 4.10.2. Protein Oxidation by Carbonyl Assay

Tissue samples were incubated with 2,4-dinitrophenylhydrazine (10 mM) in a 2.5 M HCl solution for 1 h in the dark. The samples were vortexed every 15 min. Subsequently, a 20% trichloroacetic acid (*w*/*v*) solution was added, and the solution was incubated on ice for 10 min and centrifuged for 5 min at 1000× *g* to collect protein precipitates. An additional wash was performed with 10% trichloroacetic acid (*w*/*v*). The pellet was washed three times with ethanolethyl acetate (1:1) (*v*/*v*). The final precipitates were dissolved in 6 M guanidine hydrochloride solution and incubated for 10 min at 37 °C, and the absorbance was measured at 360 nm [42].

### 4.11. Glutathione Redox Balance

To determine oxidized and reduced glutathione concentration, the tissue was deproteinized with 2 mol/L perchloric acid, centrifuged during 10 min at 1000× *g*, and the supernatant was neutralized with 2 mol/L of potassium hydroxide. The reaction medium contained 100 mmol/L of phosphate buffer (pH 7.2), 2 mmol/L of nicotinamide dinucleotide phosphate acid, 0.2 U/mL of glutathione reductase, and 70 mmol/L of 5,5′-dithiobis (2-nitrobenzoic acid). To determine the reduced glutathione, the supernatant was neutralized with 2 mol/L of potassium hydroxide, to react with 70 mmol/L of 5,5′-dithiobis (2-nitrobenzoic acid), and the absorbance values measured at 420 nm [43].

### 4.12. Antioxidant Enzyme Activities

The catalase (CAT) activity was determined by measuring the decrease in H_2_O_2_ absorbance at 240 nm. The CAT was expressed as micromoles of H_2_O_2_ reduced per minute per milligram of protein [44]. The glutathione peroxidase (GPx) activity was expressed as nanomoles of peroxide per hydroperoxide reduced per minute per milligram of protein, based on consumption of reduced nicotinamide-adenine dinucleotide phosphate at 480 nm [45].

### 4.13. Total Antioxidant Capacity (TRAP) Assay

The TRAP was measured using 2,2-azo-bis(2-amidinopropane) (ABAP, a source of alkyl peroxyl free radicals) and luminol. A mixture consisting of 20 mmol/L ABAP, 40 μmol/L luminol, and 50 mmol/L phosphate buffer (pH 7.4) was incubated to achieve a steady-state luminescence from the free radical-mediated luminol oxidation. A calibration curve was obtained by using different concentrations (between 0.2 and 1 μmol/L) of Trolox (hydrosoluble form of vitamin E). The luminescence was measured in a liquid scintillation counter using the out-of-coincidence mode, and the results were expressed in units of Trolox per milligram protein [46].

### 4.14. Statistical Analysis

The data were reported as mean ± standard error of the mean (SEM). The Kolmogorov–Smirnov normality test was used to identify parametric or non-parametric variables. One-way ANOVA was used, followed by the post hoc Student–Newman–Keuls test to determine the difference between the three groups (*n* = 6–8/each group). The repeated measures of ANOVA were used to compare the groups at different time periods (*n* = 6–8/each group). Associations between the variables were tested using Pearson correlation (*n* = 5–6/each group); *p* < 0.05 values were considered significant.

## 5. Conclusions

In conclusion, although insulin is still the main form of treatment for type 1 diabetic patients, our study shows that the treatment with insulin alone for 30 days is not able to prevent all the cardiac and autonomic alterations due to hyperglycemic state in STZ-induced diabetic rats. Clinical parameters such as HR and MAP are not altered in insulin-treated diabetic animals; however, other markers of cardiovascular mortality risk in patients such as SAP variability and baroreflex sensitivity are altered, followed by diastolic dysfunction. Our findings point to the importance of monitoring variables other than AP and HR in the cardiovascular evaluation in patients with type 1 diabetes, such as baroreflex sensitivity, AP variability, and echocardiogram. They may be clinically carried out, providing more accurate information about cardiovascular risk and possible complications associated with diabetes. It is worth emphasizing that our findings for the STZ-induced diabetic model reinforce the need for other therapies to be used in combination with insulin treatment in the management of cardiovascular risk in patients with type 1 diabetes.

## Figures and Tables

**Table 1:** Glycemia, insulin levels, and body mass evaluations of control group (C), diabetic group (D), and diabetic group treated with insulin (DI).

	C	D	DI
**Glycemia (mg/dL)**			
Initial	121 ± 6.9	370 ± 21.4 *	392 ± 22.8 *
First week	125 ± 8.3	411 ± 45.5 *	153 ± 14.9 ^#‡^
Second week	156 ± 17.8	488 ± 33.1 *	135 ± 19.9 ^#‡^
Third week	127 ± 5.3	471 ± 25.8 *	113 ± 18.3 ^#‡^
Fourth week	112 ± 8.1	456 ± 44.6 *	103 ± 15.9 ^#‡^
**Insulin (ng/mL)**			
Fourth week	0.69 ± 0.08	0.13 ± 0.02 *	1.64 ± 0.71
**Body mass (g)**			
Initial	265 ± 6.0	264 ± 6.3	268 ± 4.8
First week	297 ± 6.4	259 ± 12.3 *	304 ± 5.8 ^#‡^
Second week	312 ± 7.1 ^‡^	274 ± 14.5 *	336 ± 6.2 ^#‡¥^
Third week	335 ± 10.1 ^‡¥$^	278 ± 16.6 *	371 ± 4.6 ^#‡¥$^
Fourth week	346 ± 10.1 ^‡¥$^	279 ± 18.9 *	383 ± 5.3 ^#‡¥$†^
Mass gain	80.7 ± 10.0	15.4 ± 17.9 *	115.0 ± 6.0 ^#^

Data represented as mean ± SEM and analyzed using repeated ANOVA measurements, followed by Tukey’s post hoc test (*n* = 6–8/group). * *p* < 0.05 vs. C, ^#^ *p* < 0.05 vs. D, ^‡^ *p* < 0.05 vs. Initial. ^¥^ *p* < 0.05 vs. First week, ^$^ *p* < 0.05 vs. Second week and ^†^ *p* < 0.05 vs. Third week.

**Table 2:** Echocardiographic parameters in control group (C), diabetic group (D), and diabetic group treated with insulin (DI).

	C	D	DI
**Morphometric**			
LVEDD (cm)	0.61 ± 0.04	0.64 ± 0.04	0.68 ± 0.03
LVPWd (cm)	0.119 ± 0.003	0.096 ± 0.007 *	0.124 ± 0.004 ^#^
RWT	0.40 ± 0.01	0.30 ± 0.02 *	0.38 ± 0.01 ^#^
LV mass (g)	1.04 ± 0.04	0.82 ± 0.03 *	1.04 ± 0.03 ^#^
**Systolic function**			
Ejection fraction (%)	81.62 ± 2.10	84.78 ± 2.31	79.03 ± 2.81
Cardiac output (mL/min)	149 ± 18	126 ± 13	176 ± 10 ^#^
Stroke volume (mL)	0.48 ± 0.04	0.42 ± 0.03	0.57 ± 0.02 ^#^
**Diastolic function**			
IVRT (ms)	20.00 ± 2.24	24.17 ± 1.68	22.75 ± 1.48
IVRT normalized by RR	1.29 ± 0.11	1.68 ± 0.11 *	1.60 ± 0.08 *
E-wave (m/s)	0.79 ± 0.04	0.71 ± 0.04	0.76 ± 0.03
A-wave (m/s)	0.36 ± 0.05	0.48 ± 0.05	0.47 ± 0.04
E-wave deceleration time (ms)	51.75 ± 3.14	62.16 ± 2.44 *	60.33 ± 2.87 *
E-wave deceleration time normalized by RR	3.86 ± 0.25	4.29 ± 0.28	4.27 ± 0.22

LV end-diastolic diameter (LVEDD), left ventricular posterior wall thickness at diastole (LVPWd), relative wall thickness (RWT), left ventricle (LV), isovolumic relaxation time (IVRT), peak E-wave velocity (E-wave), peak A-wave velocity (A-wave). Data represented as mean ± SEM and analyzed by one-way ANOVA followed by post hoc Student–Newman–Keuls test (*n* = 6–8/group). * *p* < 0.05 vs. C, ^#^ *p* < 0.05 vs. D.

**Table 3:** Hemodynamic evaluations of control group (C), diabetic group (D) and diabetic group treated with insulin (DI).

	C	D	DI
MAP (mmHg)	109 ± 2	100 ± 3 *	114 ± 1 ^#^
SAP (mmHg)	122 ± 3	109 ± 3 *	125 ± 2 ^#^
DAP (mmHg)	94 ± 2	87 ± 3 *	99 ± 1 ^#^
HR (bpm)	345 ± 4	318 ± 6 *	348 ± 5 ^#^

Mean arterial pressure (MAP), systolic arterial pressure (SAP), diastolic arterial pressure (DAP), heart rate (HR). Data represented as mean ± SEM and analyzed using one-way ANOVA followed by post hoc Student–Newman–Keuls test (*n* = 6–8/group). * *p* < 0.05 vs. C, ^#^ *p* < 0.05 vs. D.

## Data Availability

The data that support the findings of this study are available from the corresponding author, K.D.A., upon reasonable request.
